# The Exploratory Burr Hole: Indication and Results at One Departmental Hospital of Benin

**DOI:** 10.1155/2013/453907

**Published:** 2013-03-21

**Authors:** Holden O. Fatigba, Alexandre S. Allodé, Kofi-M. Savi de Tové, Emile D. Mensah, Adrien M. Hodonou, Jijoho Padonou

**Affiliations:** ^1^Unit of Neurosurgery, Medicine School of Parakou University, P.O. Box 02, Borgou, Benin; ^2^Department of Surgery, Medicine School of Parakou University, Benin; ^3^Departement of Imaging Diagnose, Medicine School of Parakou University, Benin

## Abstract

*Objective*. The aim of this study was to describe the indications and results of exploratory burr hole performed at the Departmental Teaching Hospital of Borgou (Benin). *Methods*. It was a retrospective study performed from January 2008 to February 2011. It concerned patients with a closed traumatic brain injury (TBI) in which an exploratory burr hole was performed. The selection criteria were unilateral mydriasis associated with neurological deficits on the opposite side or the occurrence of a decreased consciousness associated with the appearance of a motor deficit after a lucid interval. *Results*. Amongst the 74 patients operated, 23 (31%) underwent an exploratory burr hole for which the average age was 24.8 ± 17.3 years. Sex ratio male/female was 3.6. TBI was due to road traffic accident (56.5%), a fall (26.1%), and an assault (17.4%). It was severe (47.8%), moderate (39.1%), and mild (13.1%). Mydriasis was observed in 69.6% of cases as well as neurological deficit in all patients. A lesion was observed in 15 (65.2%) cases. *Conclusion*. The exploratory burr hole seemed as an old practice, still no longer performed in full practice in Benin, and is a diagnosis and therapeutic approach. Better technical conditions would allow more relevant therapeutic options.

## 1. Introduction

Brain CT-scan and magnetic resonance imaging (MRI) had made a huge impact on diagnosis and surgical management of traumatic brain injuries [1]. Such practice is well codified and wide spread in developed countries [2, 3]. In Benin and many African countries those means of medical image still lacking. When they are in place it is either their high cost or restricted number and sometimes the fact that they are only located in big towns made them inaccessible to patients as well as to practitioners. Nevertheless TBI remains a frequent affectation in our country [4]. Even though exploratory burr hole practice is very old and abandoned or extremely limited in a modern management of TBI [3, 5–7], it is still current in countries with poor healthcare system [8–10]. The purpose of this study was to report the indications and outcomes of exploratory burr hole performed in patients with closed TBI without brain CT-scan in one underequipped hospital in Benin. 

## 2. Materials and Methods

It was a retrospective study performed between January 2008 and June 2011, at the Departmental Teaching Hospital of Borgou (Benin). This health centre is the only referral hospital in the Northern Benin. The study concerned patients who underwent an exploratory burr hole after one TBI. 

The hospital is located at 450 km from the capital city. The neurosurgery unit is hosted by a general surgery department. This department capacity is 45 beds with an admission average of 11.000 patients per year. The intensive care unit (ICU) is new and just at its beginning. There is no scanner device in the hospital, but all scans can be done in a private practice located 6 km from the hospital. 

All selected patients have a closed TBI, and none of them have had brain scan. For all cases of exploratory burr hole performed, patient selection criteria were either unilateral mydriasis associated with a motor deficit in opposite site or the occurrence of a decreasing level of consciousness associated with the appearance of a motor deficit after a lucid interval. 

Whatever the number of hole performed, the first one is always done on the temporal region and on the mydriasis side or on the opposite side of the neurological decrease after the first hole performed in temporal bone, and another one is performed in frontal or occipital bone. A maximum of three holes were performed on patient. 

## 3. Results

### 3.1. Epidemiology and Clinic

During the study period, 866 patients were admitted for TBI in which there were 199 (23%) cases of severe TBI, 103 (11.9%) cases of TBI moderate, and 564 (65.1%) cases of mild TBI. 74 (8.5%) patients has undergone surgery. The overall scanner device breakdown period during that study was of 19 months. Only 29 (39.2%) patients had a brain CT-scan, 22 (29.8%) were operated based on clinical and X-ray data and, 23 (31%) had exploratory burr hole performed without imaging. Amongst those 23 patients, 19 (82.6%) did not had brain CT-scan due to either a technical breakdown or lack of financial means, and for 4 (17.4%) their clinical state would not allow them to be moved under any circumstances. Those patients average age was of 24.8 ± 17.3 years (range from 2 years to 60 years) and accounted for 18 males and 5 females (male-female ratio 3.6). TBI that occurred in 13 (56.55%) cases was due to road traffic accidents, 6 (26.1%) to a fall, and 4 (17.4%) to an assault. Based on the initial Glasgow Coma Scale TBI was severe in 11 (47.8%) patients, moderate in 9 (39.1%), and mild in 3 (13.1%). Mydriasis observed in 16 (69.6%) cases, and the occurrence of neurological decrease in all patients was the predominant signs during examination. The symptoms or signs observed are reported in Table 1. 

Admission time to the theatre was 1.58 ± 4.14 days (range from 2 hours to 15 days). 

In 8 (34.8%) patients, decreasing level of consciousness or the appearance of a motor deficit occurred after a lucid interval. 

All patients have had an exploratory burr hole performed in the temporal location with link to other location in 15 (65.2%) cases.  Table 2 shows numbers and burr hole locations per patients. 

### 3.2. Lesion Observed and Evolution

Amongst the 23 exploratory burr hole performed, there were no apparent lesions in 8 patients (34.8%). A decompressive craniotomy was performed in those cases. An acute subdural hematoma was spotted in 7 cases (30.4%) and a haemorrhagic contusion in 2 cases (8.7%). Burr hole in these cases was converted to a craniotomy. A chronic subdural hematoma was found in 2 cases (8.7%) and an epidural hematoma in 4 cases (17.4%). Burr hole in these cases was converted to a craniotomy. The location of these lesions has been reported on Table 3. The mortality rate was of 39.1% (9/23) amongst patient whom undergone burr hole and 24.1% (7/29) amongst patients operated based on CT-scan data. The difference was not statistically significant (*P* = 0.399; *χ* ^2^ = 0.71). This mortality was of 55.6% (5/9) amongst patients for which the exploratory burr hole was negative and 26.7% (4/15) amongst patients where a surgical lesion was present. During the study period, mortality rate amongst nonoperated patients was of 6.7% (52/792); 75% of patients who died had a severe TBI. The difference in mortality rate between nonoperated dead patients and patients who had an exploratory burr hole performed was statistically significant (*P* = 0.0000017, *χ* ^2^ = 22.96). Amongst the 15 patients on whom burr hole was positive, 3 (20%) had a complete recovery after surgery. 

## 4. Discussion 

Traumatic brain injuries remain a major public health issue, and their care, a big concern in underequipped hospital [1, 11]. A Road traffic accident is the first cause and mainly occurs in male. Imaging is especially brain CT-scan input in this area, is no longer an issue, nevertheless its availability and the health system organization are still very much problematic in the third world countries. 

The occurrence of unilateral mydriasis and the first of second appearance of motor deficit are an urgent indication of needs for brain CT-scan [1, 12]. The lack of scanner or the impossibility to have or do some for whatever reasons revealed the only opportunity in performing exploratory burr hole for which outcomes are well known [3, 5, 13]. Even though, exploratory burr hole is still frequently performed, it is not a systematic therapeutic choice in our practice; it is just an illustration of our difficulties concerning TBI management in satisfactory manners [14]. This situation is similar to the one reported by Natarajan et al. [9] in 1989 in India or Viswanathan et al. [10] in Ethiopian in 2008. Performing exploratory burr hole in such condition is the outcome of serious discussion in which the neurosurgeon is always on the frontline. Without being abusive, exploratory burr hole is becoming a well justified alternative in underequipped medical centre, but reasons are opposed by Nelson [6] and Rinker et al. [7], Smith et al. [13], or Springer and Baker [15] for whom the choice of performing exploratory burr hole was due to exceptional circumstances; time of having a scan done or transferring the patients towards a specialized centre was more harmful due to their clinical state. 

**Figure 1:**
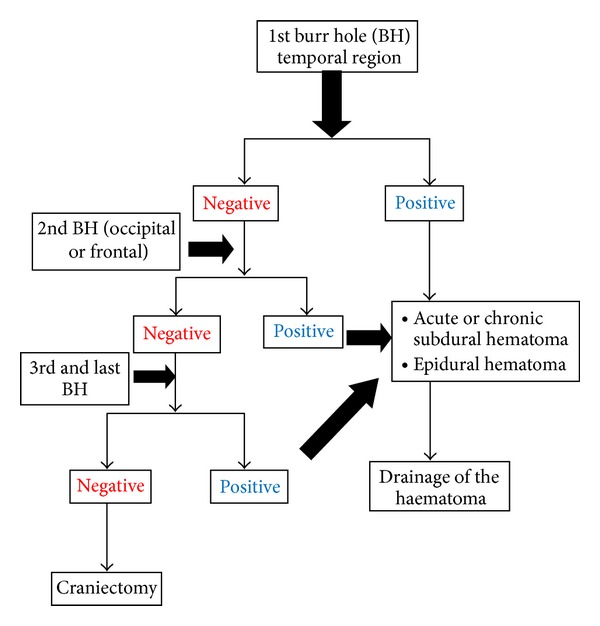
Flow diagram indicate what the BH showed and what was done with the information.

Based on diagnostic, our exploratory burr hole performances were positive in 60.9% of cases where Natarajan et al. [9] reported 55.4% in their series. This good rate is due to our rigorous selection criteria. Our therapeutic procedure strategy is summarized in Figure 1. But the exploratory burr hole based exclusively on clinical signs may create a bias in patient recruitment. Not having a brain CT-scan can create an underestimation of traumatic neurosurgical lesions; the diagnosis orientation of the examination signs can be very complex when the lesions are bilateral. On the therapeutic point of view, the exploratory burr hole had a salvation affect as indicated by the significant difference in mortality rate between patients in whom the exploration had shown a lesion and for whom it was negative. But, by taking into account all patients on whom an exploratory burr hole was performed, the overall mortality rate was higher compared to patients operated with CT-scan data. Nevertheless, brain CT-scan still very needed whatever the therapeutic choice [3, 8]. The observed mortality was due to the severity of injury as well as to the lack of knowledge associated with intracranial lesions. The insignificant difference in the mortality rate observed amongst patients operated with or without brain CT-scan shows the rigorous selection criteria of patients on who exploratory burr hole were performed. 

In our study the impact of the delay of the burr hole reported by Smith et al. [13] and Stuart et al. [16] on mortality was not assessed. Whatever, the motif and the outcomes, exploratory burr hole performed in underequipped or inadequate centre is a call upon our political leaders and decision makers on the choices and health care system strategies. Major work has already been done on TBI [1–3]. Public health centre would have to be equipped with technical means such as scanners, ongoing intensive care ward, and preadmission ward for allowing them facing and coping with the growing TBI pandemic. 

The preventive measure which is already proven to be effective [17] should be taken into account in countries with low resources. Meanwhile, the focus should be drawn on adequate training and fair dispatching of neurosurgeons, radiologist, intensive care nurses, and anesthetist across the national territory. All these measures should enable exploratory burr hole performed on TBI patients to be seen as an exceptional measure rather than the first therapeutic choice in 2011.

## 5. Conclusion 

At the Departmental Teaching Hospital of Borgou in Benin, exploratory burr hole is still pretty much one of the neurosurgery in practice. The indications are precise and restricted. 

From diagnosis and therapeutic point of view it was a useful experience. But we have to keep in mind that it has to stay an exceptional practice. The main challenge faced by underequipped countries would be an efficient health system organization in place as well upgraded technical means and conditions for coping effectively with TBI which are the first mortality cause in youngsters.

## Figures and Tables

**Table 1:** Signs or symptoms seen during neurological examination of patients.

Signs or symptoms	Number	%	95% CI
Primary LOC	15	65.2	(0.46–0.85)
Secondary LOC	8	34.8	(0.15–0.54)
Headaches	12	52.2	(0.32–0.73)
Vomiting	8	34.8	(0.15–0.54)
Seizures	7	30.4	(0.12–0.5)
Aphasia	7	30.4	(0.12–0.5)
Skull vault deformation	6	26.1	(0.05–0.44)
Mydriasis	15	65.2	(0.46–0.85)
Rhinorrhea	4	17.4	(0.09–0.25)
Epistaxis	4	17.4	(0.09–0.25)
Hemiplegia or hemiparesis	23	100	

%: percentage; CI: Confidence interval; LOC: Loss of consciousness.

**Table 2:** Repartition of the burr holes site performed in the patients.

Site of burr holes			Patients
Temporal	Frontal	Occipital	*n* (%)
+	−	−	23 (100)
+	+	−	7 (30.4)
+	−	+	4 (17.4)
+	+	+	4 (17.4)

*n*: number; %: percentage; +: burr hole performed; −: burr hole not performed.

**Table 3:** Distribution of different lesions and their location.

	Acute subdural hematoma	Chronic subdural hematoma	Epidural hematoma	Hemorrhagic contusion
Temporal	2	0	1	2
Frontal	2	1	1	0
Occipital	1	0	0	0
Temporoparietal	2	1	2	0

Total	7	2	4	2
